# Primary Extramedullary Plasmacytoma of the Heart: A Rare Manifestation of Plasmacellular Tumor

**DOI:** 10.1155/2013/290849

**Published:** 2013-12-10

**Authors:** Romagnoli Andrea, Coco Irene, Fusco Armando, Dominique De Vivo, Giovanni Simonetti

**Affiliations:** Department of Diagnostic and Molecular Imaging, Interventional Radiology and Radiation Therapy, Fondazione Policlinico “Tor Vergata”, Viale Oxford 81, 00133 Rome, Italy

## Abstract

Extramedullary plasmacytoma involving the heart is extremely rare. Primary extramedullary localizations are most commonly found in the head and neck region, with no radiologic evidence of additional skeletal lesions and normal bone marrow examination, but can occur in many other locations. They rarely occur in the heart and are commonly associated with multiple myeloma diagnosis. Here, we describe a case of primary extramedullary plasmacytoma of the heart in a 62-year-old man who presented with nocturnal dyspnea, arthralgia, and weakness. The symptoms can sometimes result from the mass effect on cardiac flow. The diagnosis and management require the same range of clinical and laboratory expertise as for patients with multiple myeloma. Their management is particularly challenging due to the lack of evidence or the presence of nonspecific symptoms. The case is presented as a learning point to remember to include plasmacytic tumors in the differential diagnosis of anaplastic tumors, even in unusual locations, such as the heart.

## 1. Introduction

Plasma cell neoplasms can present clinically in a heterogeneous fashion. Multiple myeloma (MM), the most common form, is a bone marrow-based, disseminated neoplasm that is usually associated with a wide spectrum of clinical, laboratory, and radiologic abnormalities. Uncommonly, a plasma cell neoplasm can be localized and is known as primary extramedullary plasmacytoma (EMP), with no radiologic evidence of additional skeletal lesions and normal bone marrow examination [1] presenting diagnostic challenges because of its unusual location and nonspecific or absent symptoms, but it may be present manifestation of multiple myeloma (secondary EMP).

Primary EMP develops due to uncontrolled plasma cell proliferation and monoclonal plasmacytic infiltration [2]. The more common form is solitary osseous plasmacytoma, without an increase in plasma cells in random bone marrow samples. The other form of EMP occurs at extraskeletal sites commonly in the head and neck, with normal bone marrow biopsies and skeletal radiographs [3].

Primary localization in the heart is an extremely rare malignant neoplasm of the soft tissues representing 3% of plasma cell neoplasms and less than 1% of all head and neck malignancies [4].

The symptoms can sometimes result from the mass effect on cardiac flow. The diagnosis and management require the same range of clinical and laboratory expertise as for patients with MM [5]. The treatment of these patients required a close cooperation between cancer, radiotherapy specialists, and hematologists that cooperation was crucial for planning optimum care. The present study reports a case of primary cardiac plasmacytoma in a 62-year-old male patient.

## 2. Case Report

A 62-year-old male, nonsmoker, was admitted to the “Tor Vergata University Hospital” of Rome, Italy, because of an increasing paroxysmal nocturnal dyspnea, arthralgia, and weakness. Physical examination revealed no significant hemodynamic derangement with a pulmonary ejection murmur and accentuated pulmonic second sound. Electrocardiography was only significant for sinus tachycardia. No lymphadenopathies were found on the neck and in axillae and groins.

Serum immunoglobulin quantitation showed minimal increased IgG (1398 mg/dL versus normal 543–1360 mg/dL) and IgA (280 mg/dL versus normal 58–259 mg/dL). The urine protein was normal 115 mg/24 h (normal: 0–150). Other laboratory tests showed erythrocyte sedimentation rate to be 13 mm/h (normal: 0–15) and C reactive protein to be 0.6 mg/dL (normal: <0.9).

Chest radiography demonstrated a slightly enlarged cardiac silhouette with signs of pulmonary hypertension and congestion with a bilateral small pleural effusion.

A transthoracic echocardiogram (TTE) revealed a large right atrial mass with minimal pericardial effusion. Left and right ventricular functions were within normal ranges, and both aortic and mitral valvular leaflets were focally calcified with no insufficiency or stenosis.

**Figure 1:**
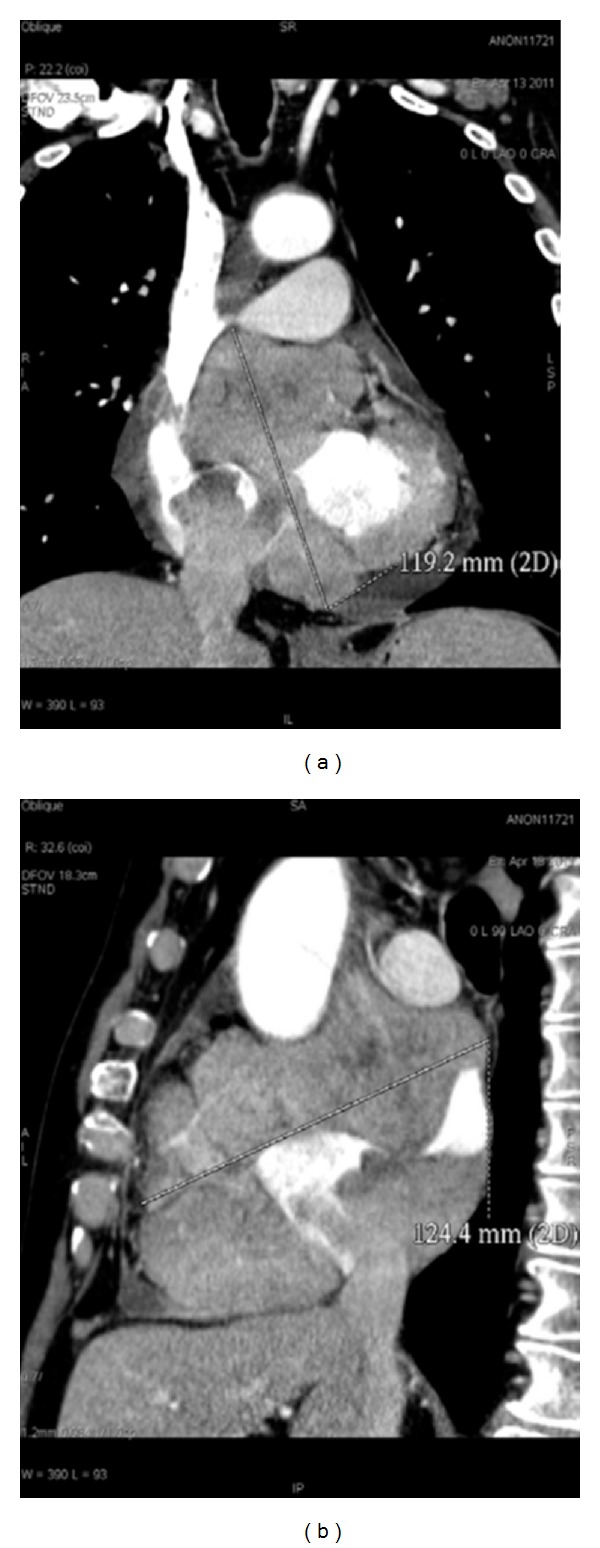
(a) Coronal reconstruction; (b) sagittal reconstruction. CT shows the presence of a 119.2 mm × 124.4 mm right atrium myocardial mass, involving the auricle and the atrial septum.

**Figure 2:**
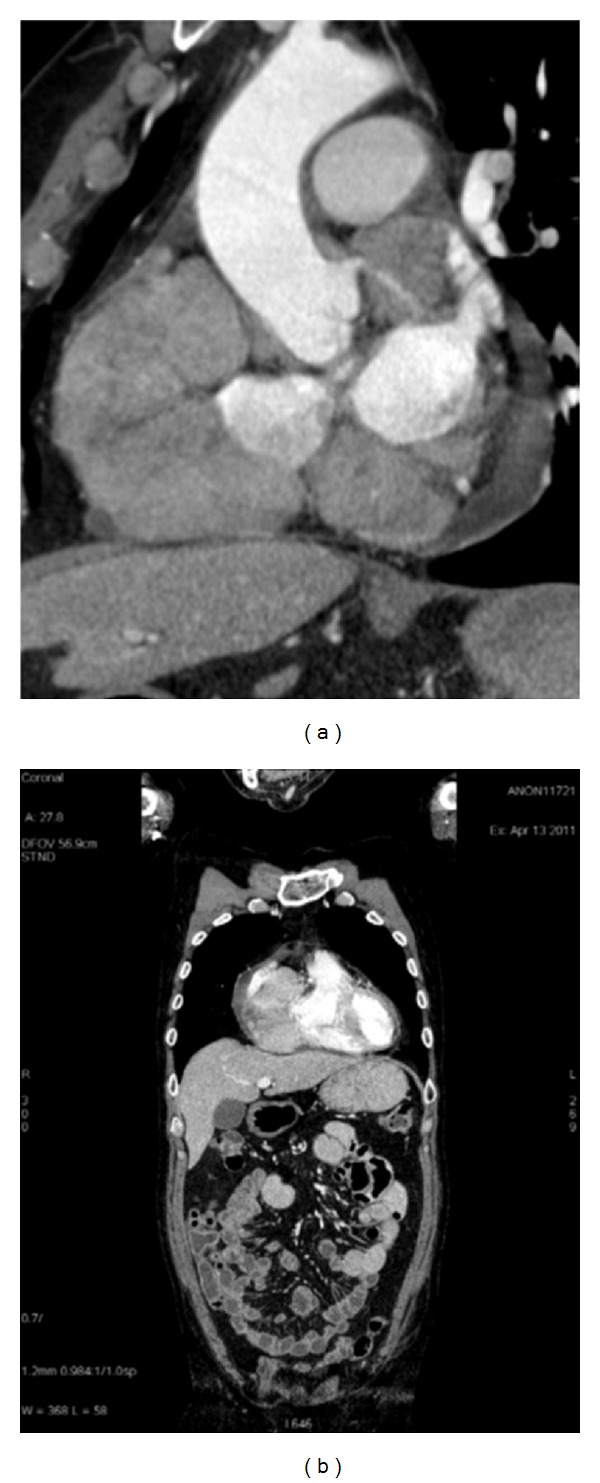
(a) Sagittal reconstruction; (b) coronal reconstruction. CT shows the presence of right atrium myocardial mass and no other abnormality in other organs.

A multislice computed tomography (CT) exam with contrast media confirmed the presence of a 3.2 cm × 2.8 cm right atrium myocardial mass, involving the auricle and the atrial septum (Figures 1 and 2). No enlarged lymph nodes were present in the mediastinum, no osteolytic bone lesions were noted in thoracic and lumbar vertebrae, and no other abnormalities were observed in other organs.

The clinical and diagnostic imaging findings suggested an occult primary malignancy with metastases to the heart, but subsequent clinical investigations revealed no primary malignancies. The transesophageal echocardiography (TEE) confirmed the presence of a large dishomogeneous myocardial mass involving the endocardial surface which was successfully aspirated with the EUS-FNA echoendoscope. The cytology of the right atrial mass revealed a hypercellular malignant neoplasm with some atypical polyhedral cells consistent with immature plasmacytoid cells suspicious for EMP or MM. Immunohistochemical assays demonstrated positive expression for the antibodies CD138, kappa chain clonality, and epithelial membrane antigen (EMA) with plasmacytoma features. The iliac bone marrow biopsy was normal, and no increased lymphocytes and plasma cells were seen. No osteolytic bone lesions were detected. A normal level of monoclonal protein in serum and urinary (24 h urine sample) electrophoresis with immunofixation confirmed the absence of occult disease elsewhere. Bence Jones protein test was negative. The absence of lymphadenopathy and hepatosplenomegaly helped to rule out a B-cell lymphoma. A hybrid positron emission tomography- (PET-) computed tomography (CT) scan showed an abnormal uptake in the expected myocardial location, and diagnosis of primary EMP of the heart was made. The patient underwent a treatment with 45 Gy of localized radiation for five consecutive weeks with high-dose corticosteroids. The mass dimension suggested the clinical necessity of supporting the radiotherapy with a consolidation chemotherapy regimen.

## 3. Discussion

Primary cardiac involvement in plasma cellular tumor is uncommon, and only a few cases with clinical significance have been reported. Primary EMP is an exceedingly rare neoplasm, and only few cases involving the atria have been encountered in the English-language literature [2]. Interestingly, all cases involved one atrium or both atria, presenting as large space-occupying tumors, causing cardiovascular compromise. Pericardial effusion also developed in some cases and caused cardiovascular dysfunction.

Diagnosis of EMP requires histologic and immune histochemical confirmations and an absence of MM, solitary osseous plasmacytoma, and plasmacytoma arising in other anatomic locations.

The tumor is composed of a monoclonal proliferation of plasma cells that may show cytological atypia mimicking a large cell lymphoma, poorly differentiated carcinoma, extramedullary myeloid cell tumor [6], or melanoma.

Myxoma, metastatic malignancies other than plasmacytoma, or less frequently sarcomas are much more commonly seen in the heart than plasmacytoma. As a result, the differential diagnosis may be overlooked unless a pathologist has a high degree of suspicion [5].

Probably an MRI mediastinum study with ECG synchronized sequences could help us to exactly detect the relationships with the myocardium, but a heart assessment was performed by TTE to evaluate cardiac function, so MRI in this case should not add any further information for improving the therapeutical approach. The only clinical findings and conventional imaging studies usually performed for the MM diagnosis did not draw exactly the patient pathologic outline.

Only the CT study gave us the correct information about the solitary presentation of the plasmacytoma, and at the same time we were able to understand the anatomic relationship with the heart.

The PET-CT study was above all important to study the active lesion metabolism and also to confirm the CT finding of solitary extramedullary mass.

This is a very important aspect considering the treatment choice. In this case the patient underwent radiotherapy, and the mass dimensions (very well obtained by multiplanar CT reconstruction) suggested introducing the patient to chemotherapy too.

## 4. Conclusions

The CT role in the diagnosis of solitary EMP in our experience was fundamental to correctly diagnose this finding, influencing the patient treatment. PET-CT total body scan was useful to confirm no evidence of more foci in the whole body and gave us important information about localization, dimensions, and vascularization.

Probably an MRI mediastinum study with ECG synchronized sequences could help us to exactly detect the relationships with the myocardium, but a heart assessment was performed by TTE to evaluate cardiac function, CT study could not settle the differential diagnosis, but thanks to its feasibility, rapidity and no invasive technique, detecting the EMP was very important in our work.
